# Aberrant right subclavian artery (ARSA) presenting with esophageal pseudo-neoplastic symptoms, dyspepsia, and hemodynamic findings

**DOI:** 10.1016/j.radcr.2025.09.033

**Published:** 2025-10-04

**Authors:** Soheil Mirzaei, Zahra Motaghed

**Affiliations:** a Department of Anatomy, Faculty of Medical Sciences, Tarbiat Modares University, Tehran, Iran; b Department of Radiology, Shahid Sattari Hospital, Shahid Beheshti University of Medical Sciences, Tehran, Iran

**Keywords:** Aberrant right subclavian artery (ARSA), Dysphagia lusoria, Esophageal compression, CT angiography

## Abstract

Aberrant right subclavian artery (ARSA) is the most common congenital anomaly of the aortic arch. Though often asymptomatic, it may cause esophageal compression and mimic neoplastic lesions. Accurate diagnosis via CT angiography is essential, especially before thoracic or cervical surgery. A 68-year-old woman presented with dyspepsia, dyspnea, and epigastric pain. Endoscopy revealed a polypoid lesion suggestive of malignancy. CT imaging showed ARSA passing posterior to the esophagus, with no mass detected. Hemodynamic evaluation revealed inter-arm blood pressure discrepancy and bradycardia. ARSA can lead to dysphagia lusoria and other compressive symptoms. Its association with Kommerell’s diverticulum and non-recurrent laryngeal nerve has surgical implications. Treatment options include open, endovascular, and hybrid approaches. ARSA should be considered in patients with atypical esophageal symptoms. Imaging plays a key role in diagnosis and guiding appropriate, individualized treatment.

## Introduction

Aberrant right subclavian artery (ARSA) is the most common congenital anomaly of the aortic arch and is frequently associated with cardiovascular malformations such as tetralogy of Fallot, transposition of the great arteries, ventricular and atrial septal defects, as well as genetic syndromes including Down, DiGeorge, and Edwards syndromes [[1], [2], [3], [4]]. ARSA typically courses posterior to the esophagus and trachea, and depending on its size and trajectory, may cause significant compression of these structures [5,4].

From an embryological perspective, ARSA results from regression of the proximal segment of the right fourth aortic arch. In the absence of a brachiocephalic trunk, the artery arises directly from the dorsal aorta and continues toward the right upper extremity by passing behind the esophagus. This pathway is explained within the framework of Edwards’ double arch hypothesis [2,6,7].

Additionally, ARSA may be associated with anomalies such as a common carotid trunk, Kommerell’s diverticulum, thyroid ima artery, aberrant origin of the internal mammary artery, non-recurrent right laryngeal nerve, and a right-sided aortic arch [1].

### Prevalence

ARSA is the most common congenital anomaly of the aortic arch, often incidentally diagnosed during imaging. Its prevalence ranges from 0.1% to 4.4%, with regional variations reported: Europe 0.11%-0.36%, Asia 0.1%-0.2%, America 0.5%, and New Zealand 0.8% [1,3,5,[7], [8], [9],]. In most cases (80–85%), ARSA courses posterior to the esophagus; however, in some individuals it is located between the trachea and esophagus (12.7%-15%) or anterior to the trachea (4.2%-5%) [10,1,6]. Approximately 14.9%-60% of ARSA cases originate from a Kommerell diverticulum, a remnant of the right dorsal aorta observed as a localized dilation at the origin site [10]. The presence of a non-recurrent right laryngeal nerve—directly entering the larynx from the cervical vagus trunk—is also considered a strong indicator of ARSA, with a prevalence of 0.3%-1.6% [2,10].

### Symptoms

ARSA is typically asymptomatic until the fourth or fifth decade of life. In symptomatic cases, it may cause tracheal or esophageal compression, known as ``arteria lusoria'' or ``dysphagia lusoria'' [1,4,5,9]. The most frequent clinical symptom is dysphagia, observed in approximately 71% of patients, particularly when ARSA has a posterior course relative to the esophagus [1,9,10]. Other symptoms include dyspnea (18%), retrosternal pain, cough, significant weight loss, recurrent pulmonary infections, epigastric pain, back pain, and numbness of the right upper extremity—especially when the artery courses posterior to the trachea [1,10,11]. Studies have demonstrated that increased cross-sectional area of ARSA near the esophagus and reduced distance to the trachea are strongly correlated with the development of dysphagia [1]. Less common manifestations include recurrent laryngeal nerve palsy, claudication, and subclavian steal syndrome [11].

## Case presentation

### Patient history

A 68-year-old woman presented to the gastroenterology clinic with dyspepsia, dyspnea, and severe epigastric pain. Her symptoms had progressively worsened over recent weeks and had markedly impaired her quality of life.

### Endoscopic findings

Upper endoscopy revealed a smooth, pulsatile polypoid lesion on the posterior esophageal wall, located 28 cm from the incisors. The endoscopic appearance raised suspicion for a submucosal or vascular anomaly rather than a neoplastic process. Due to the pulsatile nature of the lesion and intact mucosal surface, biopsy was deferred to avoid potential hemorrhage. Although ARSA had not yet been considered at that stage, the atypical vascular morphology prompted caution during endoscopic evaluation.

### Imaging studies

**Fig. 1:**
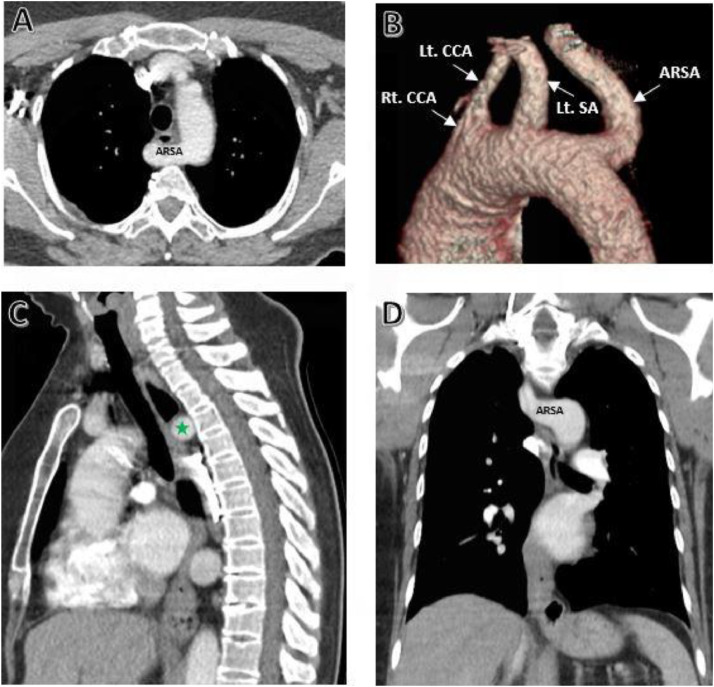
Contrast-enhanced CT scan images demonstrating the aberrant course of the right subclavian artery passing posterior to the mid-esophageal segment (green star). This vascular trajectory is consistent with the typical appearance of a right-sided aortic arch and aberrant right subclavian artery (ARSA). Image views: A) Axial B) Three-dimensional reconstruction C) Sagittal D) Coronal - ARSA, aberrant right subclavian artery; Rt. CCA, right common carotid artery; Lt. CCA, left common carotid artery; Lt. SA, left subclavian artery.

A contrast-enhanced chest CT scan was performed. Three-dimensional reconstructions clearly demonstrated an ARSA coursing posterior to the esophagus and continuing toward the right hemithorax. Despite this anatomic deviation, there was no evidence of an intraluminal esophageal mass or a space-occupying lesion on imaging. At the gastroesophageal junction (GEJ), mural thickening was observed in association with a hiatal hernia, which could contribute to the patient’s gastrointestinal symptoms. Notably, there was marked tortuosity of the descending aorta, potentially reflecting age-related structural changes or hemodynamic stress (Fig. 1).

### Hemodynamic assessment

Vital signs showed a heart rate of 58 beats per minute, which may be clinically relevant given the patient’s age and presentation. A significant inter-arm blood pressure difference was recorded: 128/81 mmHg in the right arm and 109/72 mmHg in the left arm. This discrepancy could be attributable to the aberrant course of the left subclavian artery, particularly when a retroesophageal trajectory alters blood flow and peripheral vascular resistance. In cases of ARSA, such inter-limb blood pressure differences may serve as an adjunctive diagnostic finding.

## Clinical discussion

ARSA typically arises as the fourth and most distal branch of the aortic arch [5]. This congenital anomaly usually originates from the descending thoracic aorta and courses posterior to the esophagus in more than 80% of cases [7,9]. The passage of ARSA around the trachea and esophagus may form an incomplete vascular ring, potentially leading to significant compression, dysphagia, pressure-induced necrosis, and in severe cases, the development of an arterioesophageal fistula [10].

In many instances, ARSA is associated with Kommerell’s diverticulum, a remnant of the embryonic fourth aortic arch, which carries a risk of aneurysmal degeneration and rupture. Management of this complex anatomical configuration remains challenging [5,11]. Furthermore, the relationship between ARSA and the non-recurrent inferior laryngeal nerve (NRILN) holds considerable surgical importance, particularly in esophageal cancer cases. This nerve originates directly from the cervical vagus trunk and is anatomically linked to the aberrant course of ARSA [2].

ARSA may also be associated with genetic syndromes such as Down, Edwards, Patau, Turner, DiGeorge, Noonan, postnatal rubella, and Potter syndrome [10]. These anomalies are critical in surgical planning, as they may predispose patients to postoperative complications such as subclavian steal syndrome [8,9].

Although ARSA is the most common anomaly of the aortic arch, it is frequently identified incidentally during routine imaging modalities such as CT, MR angiography, color Doppler ultrasound, X-ray, endoscopy, and barium swallow studies. These techniques are particularly effective in detecting pulsatile external compression of the esophagus [1,7,9,10].

Preoperative identification of ARSA is crucial prior to thyroid and parathyroid surgeries, carotid endarterectomy, cervical procedures, and endovascular interventions, as this anomaly is susceptible to iatrogenic injury [1,2,6,7,10]. Understanding the origin and course of ARSA can help prevent life-threatening hemorrhage and injury to the non-recurrent inferior laryngeal nerve (NRILN). Although this nerve is not directly visualized preoperatively, the presence of ARSA on CT imaging strongly suggests its existence, underscoring the importance of contrast-enhanced preoperative imaging in preserving this vital structure [1,2,10].

Various studies have reported ARSA as an anatomical anomaly with an estimated prevalence of approximately 2.2%. It is more commonly observed in females and typically courses posterior to the esophagus. Associated findings such as Kommerell’s diverticulum and the non-recurrent inferior laryngeal nerve have been frequently documented [10].

Cases of ARSA coexisting with complex cardiovascular anomalies—including bicuspid aortic valve, persistent right superior vena cava, ventricular septal defect, and common carotid trunk—have also been described, even in patients without compressive symptoms [1,6]. Surgical correction strategies have included open procedures and hybrid techniques, such as ligation of the ARSA origin and reimplantation into the carotid artery without grafting, yielding favorable outcomes [5].

In esophageal surgery, thoracoscopic semi-prone esophagectomy and mediastinoscopic approaches have been successfully performed in patients with esophageal cancer and concurrent ARSA, allowing for preservation of the non-recurrent laryngeal nerve and precise lymphadenectomy [2,8]. Several studies emphasize that preoperative identification of ARSA can prevent serious complications such as catastrophic hemorrhage or nerve injury [7].

In symptomatic patients with esophageal compression, a bypass to the ascending aorta has proven effective, with anesthetic management playing a critical role in therapeutic success [9]. A combined technique involving carotid-to-subclavian bypass and endovascular occlusion of the diverticulum has also been proposed for complex ARSA cases, demonstrating positive outcomes [11].

Ultimately, advanced imaging modalities such as CT angiography serve as essential tools for accurately delineating the course of ARSA and guiding appropriate treatment strategies in symptomatic patients [4].

Although many aortic arch variants are asymptomatic and do not require intervention, surgical treatment is recommended in symptomatic patients presenting with dysphagia, dyspnea, or chronic cough—even in the presence of a small Kommerell’s diverticulum [6,11].

Three primary approaches have been described for the correction of ARSA: open surgery, endovascular repair using thoracic endovascular aortic repair (TEVAR), and hybrid techniques. Among open surgical methods, right thoracotomy is the most commonly employed approach, although median sternotomy or video-assisted thoracoscopic surgery (VATS) may be utilized in select cases. In anatomically complex scenarios, aortic arch reconstruction with hypothermic circulatory arrest may be necessary, which is associated with prolonged hospitalization and increased risk of postoperative pneumonia.

TEVAR involves occlusion of the ARSA origin and relies on collateral circulation; however, it is generally less favored in younger patients due to long-term considerations. The hybrid technique combines endovascular occlusion of the ARSA with a carotid-to-subclavian bypass via a supraclavicular incision, effectively maintaining perfusion and alleviating esophageal compression.

Treatment selection should be individualized based on patient anatomy, clinical symptoms, and the absence of standardized guidelines [5]. Additionally, thoracic paravertebral block has been shown to provide effective and safe analgesia in procedures such as thoracotomy [9]. In certain cases, semi-prone thoracoscopic esophagectomy has been proposed as a minimally invasive and effective strategy for managing ARSA [2].

In the present case, following the diagnosis of ARSA via contrast-enhanced CT angiography, conservative management was pursued due to the absence of dysphagia or compressive symptoms. The patient was placed under clinical observation, and no surgical or endovascular intervention was undertaken. This approach was deemed appropriate given the lack of Kommerell’s diverticulum or aneurysmal changes.

Furthermore, several reports have documented cases in which an ARSA was initially misinterpreted as an esophageal mass, underscoring the diagnostic challenges and clinical relevance of this anomaly. Brauner et al. [12] described a case of esophageal foreign body impaction caused by external compression from ARSA, which necessitated urgent intervention. Similarly, Howey et al. [13] reported a patient presenting with dysphagia and retrosternal discomfort, initially attributed to hiatal hernia, but later diagnosed with ARSA. These cases highlight the potential for ARSA to mimic submucosal or neoplastic esophageal lesions and emphasize the importance of accurate preoperative identification to avoid unnecessary diagnostic procedures or interventions.

## Conclusion

In conclusion, ARSA, despite its relatively high prevalence, can present with gastrointestinal and neoplastic-like symptoms, making its diagnosis challenging. Accurate identification of this anomaly through advanced imaging techniques, especially in symptomatic patients, plays a crucial role in preventing surgical complications and guiding appropriate therapeutic strategies. The hemodynamic findings and anatomical relationship of ARSA with vital structures such as the non-recurrent laryngeal nerve further underscore its clinical significance. A personalized treatment approach—whether open surgery, endovascular intervention, or hybrid techniques—can lead to symptom relief and reduce potential risks.

## Patient consent

Written informed consent was obtained from the patient for publication of this case report and accompanying images. A copy of the written consent is available for review by the Editor-in-Chief of this journal on request.
